# When those who know *do* share: Group goals facilitate information sharing, but social power does not undermine it

**DOI:** 10.1371/journal.pone.0213795

**Published:** 2019-03-11

**Authors:** Annika Scholl, Florian Landkammer, Kai Sassenberg

**Affiliations:** 1 Leibniz-Institut für Wissensmedien, Tuebingen, Germany; 2 University of Tuebingen, Tuebingen, Germany; Ghent University, BELGIUM

## Abstract

Good team decisions require that team members share information with each other. Yet, members often tend to selfishly withhold important information. Does this tendency depend on their power within the team? Power-holders frequently act more selfishly (than the powerless)—accordingly, they might be tempted to withhold information. We predicted that given a *task* goal to ‘solve a task’, power-holders would selfishly share less information than the powerless. However, a *group* goal to ‘solve the task together’ would compensate for this selfishness, heightening particularly power-holders’ information sharing. In parallel, an *individual* goal to ‘solve the task alone’ may heighten selfishness and lower information sharing (even) among the powerless. We report five experiments (*N* = 1305), comprising all studies conducted in their original order. Analyses yielded weak to no evidence for these predictions; the findings rather supported the beneficial role of a *group goal* to ensure information sharing for both the powerful and the powerless.

## Introduction

Imagine a team leader and her assistants making decisions together in a team. This could be a business team discussing the best proposal for a new client or a group of doctors deciding upon the ideal treatment plan for a patient. In such contexts, each team member may have access to unique, important pieces of information—information that the other members do not know about. If the leader and all members share this information with each other, the team can reach an optimal decision together. Yet, at times, team members do not work together well. Rather, each member often selfishly tries to find the solution *alone*, by oneself—be it to be the best in the team, to maintain status, or for other selfish reasons [1,2]. To achieve this, rather than sharing information with each other, team members tend to keep information to themselves (e.g., [1, 3, 4, 5]). This can cause suboptimal group decisions with potentially high costs—such as, in the examples above, a failing proposal or suboptimal treatment plan (for a meta-analysis, see [6]). It is, thus, important to understand when team members do (not) selfishly refrain from sharing their information with each other.

In team decision-making, the team leader plays an important role (e.g., by asking questions in group discussions [7]). Just as team members, leaders may have access to unique and, thus, important pieces of information they can share or withhold. In the examples illustrated above, however, will the leader share her information? Or will she withhold her information to identify the best solution on her own, without the team members? On the one hand, one could expect that especially the leader will consider it her duty to lead by example and to make sure to boost team performance (e.g., [8]). She might, thus, share all her information to facilitate an optimal team decision. Yet, on the other hand, being in such a powerful position in a team can make people selfishly neglect team interests for the sake of personal benefits (see, e.g., [9]). Accordingly, the team leader may tend to keep information to herself (more so than the less powerful team members).

The present research tested if a position of high (compared to low) social power does, indeed, reduce the willingness to share important information. Specifically, we examined whether such a potentially ‘corruptive effect’ of power on information sharing may be explained by selfish motives among the powerful. We did so in three steps, namely, by examining the role of three different *types of goals* for power-holders’ (and powerless people’s) information sharing:

First, we examined how power predicts information sharing under standard conditions. That is, we tested if under a standard *task goal* (to simply ‘solve a task’), power-holders share less information than the powerless. Second, building upon this, we then examined we investigated if a *group goal* (to perform well ‘together as a group’) can compensate for this selfishness—motivating power-holders to share more information. Third, we tested if an *individual goal* (to perform well ‘alone by oneself’) evokes selfishness even among the powerless—that is, those who usually do share—lowering information sharing. Finally, we examined if selfish motivation mediates the effects of power on information sharing. In doing so, the present research seeks to contribute to an understanding of how power relations within a group may limit information sharing and outline effective ways in overcoming this (namely, by promoting specific goals).

### Why social power may promote selfishness

Social power implies asymmetric control over one’s own and others’ situation by affording access to valued resources (e.g., financial rewards, social appreciation; [10]). This means that a power-holder has relative control over outcomes and is relatively independent from others, whereas the outcomes of the powerless largely depend on the power-holder. Being independent when being powerful activates approach tendencies and promotes disinhibited behavior; in contrast, depending on others’ resources when having low power elicits inhibition tendencies and concerns about how one is being evaluated by others ([11]). Making a slightly nuanced prediction, the Situated Focus Theory [12] suggests that their independence better enables power-holders to focus on their current—often individual—goal at hand than those low in power. In essence, both approaches suggest that power enables people to act on behalf of their personal, usually *selfish* agenda.

Indeed, social power has been shown to evoke a focus on the self—such as personal desires, interests, or opinions [12; 13]. To give some examples, compared to the powerless, power-holders are more inspired by their own than others’ contributions [14], voice their own opinions more openly [15], reflect more about their own actions [16], and competitively ignore others’ useful advice [17, 18]; similarly, the powerful can sacrifice others’ benefits for the personal goal of maintaining their power [8]. In sum, power seems to focus people more on the self and can ‘corrupt’ towards more selfish behavior.

Power-holders’ higher selfishness may have implications for their willingness to share unique, important information, such as when solving a task or making decisions in a team. Power-holders may be more motivated to keep important information that only they possess to themselves—for selfish reasons, like retaining superior status in the group, outperforming the other members, or gaining a reputation. In contrast, the powerless, being less selfish and more concerned about how others evaluate them, may more willing to share their information in the team. In the following, we refer to this prediction as potential ‘corruptive effect’ of high (versus low) power on information sharing (Hypothesis 1).

Importantly, we expect higher *selfishness* among power-holders to cause this effect. This means that such a ‘corruptive effect’ of power should only occur if current goals (indicating how to solve a task) do allow power-holders to pursue such selfish interests. Following this reasoning, the powerful may start sharing their information when they pursue a goal that *compensates* their selfishness. Similarly, even the powerless—those who usually tend to share information selflessly—should reduce their information sharing when given a goal that explicitly *promotes* selfishness. In combination, support for these predictions would show that selfishness does drive a ‘corruptive effect’ of power on information sharing. We now examine the role of such goals.

### How (group) goals alter selfish tendencies

Making decisions and solving tasks together with others in a team provides room for different motives. On the one hand, such group contexts allow members to cooperate, share, and find the best solution *together*; on the other hand, these contexts enable group members to compete, defend their preferences, and discover the best solution *alone* [1]. Indeed, by default, group members often do not cooperate with one another. Instead, some members selfishly satisfy individual goals when no specific goal *how* to solve a task is given [2]. Accordingly, given a standard *task* goal to simply ‘solve a task’ at hand, without specifying how to do so exactly, leaves room for selfishness. In a group context, such selfishness implies trying to solve the task irrespective of (prior to, or even better than) others and hinders information sharing.

As we argued above, in standard situations with a task goal to ‘solve a task’, high (versus low) power should evoke selfishness and, thereby, reduce the sharing of information that is critical for the task (corruptive effect; Hypothesis 1). As an alternative to task goals, however, members can be motivated to pursue a *group goal*—the goal to ‘perform well together as a group’. Members following group goals allocate their resources away from individual concerns towards group concerns [19]. As such, group goals offer less room for selfishness and more scope for cooperation.

Indeed, pursuing a group goal (compared to a task or individual goal) can enhance perceived cooperation within a group [20], boost information seeking among members [21], and encourage them to consider each other’s information [22]. Finally, pursuing a group goal can even be beneficial when being in a team with those people that represent competitors in other contexts [23]. Accordingly, compared to a task goal, we propose that setting a group goal will compensate for power-holders’ selfishness and heighten their information sharing. We refer to this as potential ‘compensatory effect’ of a group goal among power-holders. Initial evidence suggests that power-holders’ selfishness can, indeed, be overcome when power-holders attend to *others*, rather than personal interests—such as, when they care about others (e.g., [24,25,26,27,28]) or take over another person’s perspective [29]. Building upon this, we predict that a *group* (rather than task) goal will compensate power-holders’ selfishness in favor of cooperating with others—here, by sharing one’s important information (‘compensatory effect’; Hypothesis 2).

Another way to demonstrate that selfishness causes the effects of power under a task goal is to promote selfishness among the powerless, that is, those who are usually less selfish. This may be achieved by means of an *individual goal*. If a person follows an individual goal to perform well ‘alone by oneself, as an individual person’ (e.g., to be the first one to find the solution of a task), that person seeks to stand out individually [20]. To achieve this, it is useful to keep important information to the self, in order to prevent others from finding the solution before oneself succeeds. Power-holders, being more selfish, may often adopt and pursue such individual goals (when there is room for it, that is, under standard task goal conditions). Yet, even the powerless may share less of their information when explicitly provided with an *individual* (rather than task) goal; we refer to this prediction as the ‘selfish effect’ of an individual versus task goal among the powerless (Hypothesis 3).

**Fig 1:**
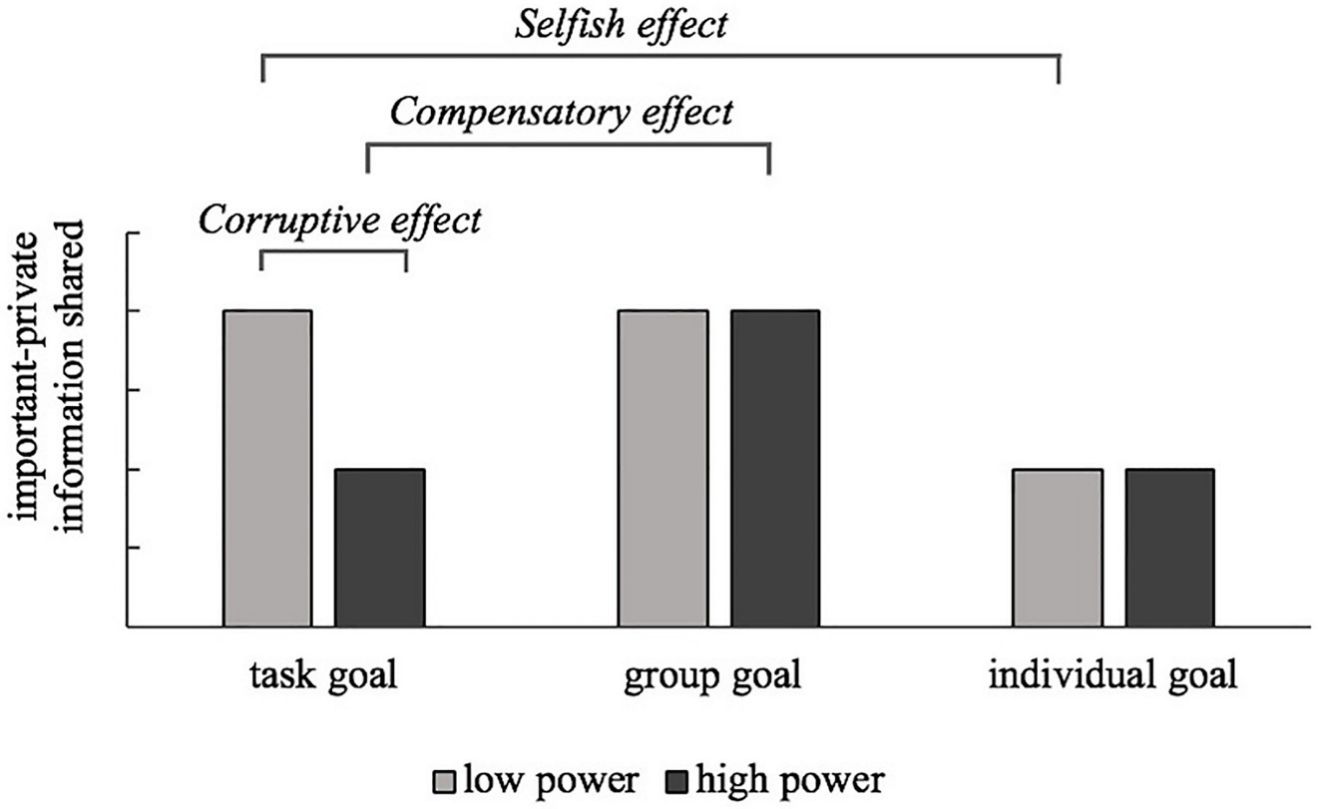
Predictions. Overview of predictions for the role of power and goals on information sharing: (1) the *corruptive effec*t of high vs. low power, (2) the *compensatory effect* of a group vs. task goal among high-power people, and (3) the *selfish effect* of an individual vs. task goal among low-power people.

Taken together, we propose that social power may diminish information sharing because of higher selfishness. We tested this by means of the following predictions: (1) In case of a *task* goal, power-holders share less information than the powerless (corruptive effect); (2) A group (versus task) goal will compensate this, promoting information sharing among the usually selfish power-holders (compensatory effect); (3) Similarly, an individual (versus task) goal will increase selfishness among the usually selfless powerless, lowering their information sharing (selfish effect). Fig 1 illustrates these predictions. Note that, these predictions, indirectly, imply the assumption that high- and low-power people share a similar amount of information under a group goal, in line with earlier findings on power effects [30, 31,32].

Finally, we expected that (4) the effects of power and goal on information sharing to be mediated by higher selfish motivation (mediation effect); however, given that we did not find evidence for the corruptive effect and very limited evidence for the selfish effect across studies, we report results for this mediation in S1 Supporting Information (and details about the measures in S2 Supporting Information).

Importantly, we expect these effects to be specific to sharing *critical* pieces of information—those pieces of information that are important to find a solution and only known to the person in question (so-called *important-private* information). We propose that effects occur only for these specific types of information, rather than unimportant or publicly available information, because withholding these critical pieces of information can best satisfy selfish tendencies (see [1]).

### The current research

In total, we conducted five experiments, reported in their original order. Experiments 1–3 set out to test predictions with ideal cell sizes of 30 participants per condition (as our standard for ideal sample size at the given time, now an outdated lab-rule). Individual results of these three experiments largely supported predictions; however, given current standards, these three experiments had relatively small samples and may have been somewhat underpowered; indeed, sensitivity analyses showed that with our given sample sizes, the analysis had 80% power (α = .05) to detect an effect size of *f* = .25 in Experiments 1 and 2, and an effect size of *f* = .21 in Experiment 3 (i.e., to detect a medium or slightly smaller than medium effect).

To address this, we (1) estimated mean effect size across these first three studies via meta-analyses and, then, (2) performed two sufficiently powered replications of Experiment 3 (namely, Experiments 4 and 5). Mean effect size across Experiments 1–3 was *r* = .18; based on this estimation, we performed a priori power analyses (*f* = .183, α = .05, (1-β) = .90; G*power; [33]) and aimed for minimum ideal sample sizes of *N* = 316 and 380 for Experiments 4 and 5 (slightly higher for Experiment 5 in case this mean effect size was, still, overestimated); both replications were preregistered at aspredicted.org (see S3 and S4 Supporting Information; preregistration was not our standard procedure at the time of conducting Experiments 1–3).

Data collection stopped once roughly the ideal sample size was reached, after which we started analyses; our sample in Experiment 5 is larger than intended because data came in unexpectedly quickly. Sensitivity analyses (G*power; [33]) revealed that for Experiment 4, with an actual sample size of *N* = 323, the analysis had 90% power to detect an effect size of *f*^2^ = .181; for Experiment 5, with an actual sample size of *N* = 547, our analysis had 90% power to detect an effect size of *f*^2^ = .139. Following our procedure from the first three experiments, we then analyzed Experiments 4 and 5 both individually and meta-analytically (combined with Experiments 1–3; data for all studies is available at https://osf.io/u9x8a/).

We report all manipulations and exclusions (if any) from data collection, and these five experiments comprise all studies we have performed in our lab so far to test the effects of power and goals on information sharing. We fully report these to allow readers to gain insights about the sum of evidence gathered on this research question and to evaluate the robustness of effects. All experiments implemented the same goal manipulation [22] and information sharing paradigm [1]. We applied two well-established power manipulations: Experiment 1 implemented assigned power roles in the lab [34] and the information sharing paradigm in a subsequent, unrelated context—to rule-out potential demand effects. Experiments 2–5 manipulated power in a controlled business simulation [35] and implemented the information sharing paradigm in that same context. The Local Ethics Committee at the Leibniz-Institut fuer Wissensmedien Tuebingen, approved all studies (no. LEK 2014/004). Participants provided written informed consent prior to their participation.

## Materials and methods: Experiments 1–5

### Participants and design

We manipulated *power* and *goal* between subjects with random assignment to condition. Experiments 1, 3, 4, and 5 used a 2 (power: high vs. low) x 3 (goal: task vs. group vs. individual) design—to test (1) the corruptive effect, the (2) compensatory effect, and (3) the selfish effect. Experiment 2 used a more parsimonious design, without individual goal condition, implementing a 2 (power: high vs. low) x 2 (goal: task vs. group goal) to test the (1) corruptive and (2) compensatory effect, which was initially our main focus. Experiments 2 and 3 additionally measured selfish motivation to test (4) the mediation effect.

*Experiment 1* took place in the lab with 127 students (83 female, *M*_age_ = 23.73) in return for 8€ (approximately 9.5 US$) as compensation. *Experiment 2* included 123 students (63 female, *M*_age_ = 22.96) participating in a paper-pencil experiment on campus in return for a chocolate bar. In *Experiment 3*, 185 individuals from a community sample (88 female, *M*_age_ = 34.30) participated in an online-experiment on Amazon Mechanical Turk. *Experiment 4* (*N* = 323, 141 female, *M*_age_ = 36.38) and *Experiment 5* (*N* = 547, 368 female, 154 male, 11 other/n.a, *M*_age_ = 22.28) were conducted online and served as highly powered replications of Experiment 3. In total, 1305 individuals participated in five experiments. ^2^

Note that demographic data from some individual participants was missing (in Experiment 2: two participants did not indicate their age; eight participants did not indicate gender. Experiment 3: five participants did not indicate age and gender. Experiment 5: nineteen participants did not provide information about gender and/or age). Study materials for Experiments 1 and 2 were, originally, designed in a way to test how naïve student participants share information in an unacquainted, experimentally controlled work scenario; accordingly, participants who *evidently* did not fulfill the basic requirement of being an undergraduate were excluded (i.e., who indicated being employed, a researcher, or in a semester of 20 and higher; that is, who had already gained experience with power at work, thus differing from the other participants; Experiment 1: five participants, Experiment 2: six participants). Including these cases in the analyses does not alter the pattern of main results; still, we excluded these cases because they did not belong to our originally intended sample. In Experiment 3, we had to exclude two participants who finished the whole survey in less than 2 minutes and, thus, could not have carefully read materials.

### Procedure and measures

We (1) manipulated power, (2) manipulated goal, (3) measured information sharing, and (4) measured selfish motivation as mediator (only Experiments 2 and 3). This procedure was identical across studies.

#### Experiment 1

As the first test of our predictions, this study manipulated power and goals in one context and measured information sharing in another (“unrelated”) context—to test for carry-over effects and rule out that our effects are driven by social desirability or demand effects. Prior to this study, participants completed a questionnaire for an unrelated study.

We manipulated power with established role assignments for an alleged collaborative task ([34]; for similar procedures see, e.g., [16]). Participants completed a “leadership questionnaire”, on which basis they were (supposedly) assigned the role of a construction manager (*high* power) or a construction worker (*low* power) for the construction of a tangram puzzle in triads; each triad involved one manager and two workers and in fact, role assignments were randomized. Managers were ‘in charge of the coordination task’ and would instruct the workers to build a tangram; they would decide how to structure the task and on which basis the results were to be evaluated. In parallel, workers were supposed to ‘execute the coordination task and follow the manager’s instructions’ to build a tangram. All participants learned that managers would finally evaluate workers in a private questionnaire, but workers would not evaluate the manager. The manager’s evaluation would determine how 15 tickets for a lottery of a 50€-voucher for an online bookseller were to be distributed in the triad; as such, power comprised asymmetric outcome control (see [34]).

We manipulated *goal* with an established procedure [22]. Participants read that the aim of the tangram task was either to ‘be aware of the correct solution’ (*task goal*), to ‘find the correct solution together as a group’ (*group goal*), or ‘to know the correct solution one-self, as a person’ (*individual goal*). While waiting for their partner to get ready for the tangram (see [16, 34]), we introduced them to a supposedly ‘unrelated second study’—which in fact measured our outcome information sharing.

Participants here completed an information sharing paradigm ([1], Experiment 3). This paradigm involves solving a riddle to find a treasure. The riddle can only be solved if group members do share information with each other—in particular, the important pieces of information that only they possess.

Introducing them to the paradigm, participants read about an old monastery, whose monk had buried a treasure in his prospective grave during the time of Reformation. To find the treasure, participants needed to combine pieces of information that the monk had distributed to *four* different letters. The first letter was published—that is, its information was *public* and known to all. Participants read that they had bought the second letter, which contained *private* information (known only to the participant), on a flea market; two other (non-specified) people possessed the third and fourth letter, which, again, contained private information known only to its respective owner.

**Fig 2:**
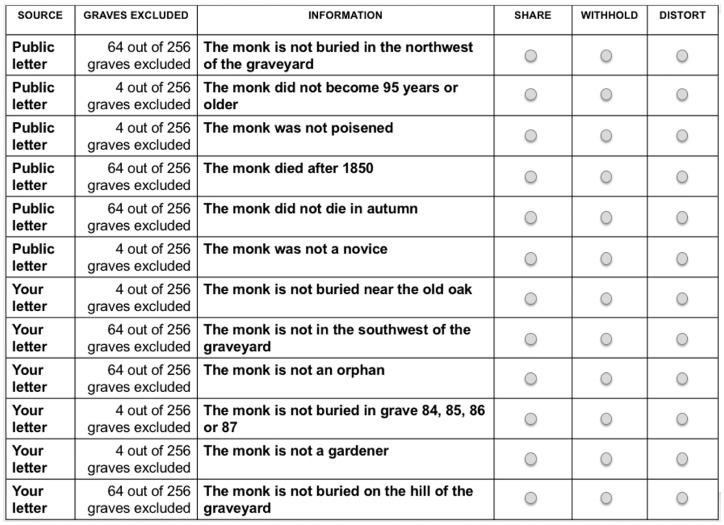
Information sharing paradigm. It included 12 pieces of information. “Source” indicates if the information was *public* (known to all; public letter) or *private* (known only to participants; your letter), “graves excluded” indicates if information was *important* (excluding many graves) or *unimportant* (excluding only few graves). Critical pieces of information are the private-important ones.

They read that each letter contained 12 pieces of information; each piece was marked as public or private and as important or unimportant (see Fig 2). Participants’ letter possessed three pieces of each category—three pieces of important-private, unimportant-private, important-public, and unimportant-public information each. To solve the riddle, participants learned that, in total, at least 18 pieces of information needed to be exchanged between themselves and the other two owners—especially the *important-private pieces* of information were needed to find the treasure. This clearly stated that one could only find the treasure by sharing one’s own information and/or by relying on the other owners to share information (because one’s own pieces of information did not suffice to identify the grave).

Participants were now about to meet the two owners of the third and fourth letter to exchange information. Preparing for this meeting, participants decided which of their 12 pieces of information they would want to share with the others, or withhold, or distort (i.e., change its content; see Fig 2). Because participants had learned that sharing *important-private* pieces of information was critical for solving the riddle, the number of these specific pieces of information they had selected to *share* represented the most sensitive indicator for (low) selfish tendencies and served as our dependent variable. After making their decisions for each piece of information, participants returned to the alleged team “tangram task”, completed a manipulation check, and exploratory measures.

For manipulation checks, participants reported to which extent they pursued a *group goal* (“My goal is to jointly find the solution for the tangram”) and indicated their *subjective power* on three items (“How much influence will you have in the following [tangram] task compared to your partner”; 1 = *I have less influence*, to 7 = *I have more influence*; “I have a leading position in the following task”; “I have a rather subordinate role in the following task”, reverse-scored; 1 = *does not apply at all*, to 7 = *completely applies*; α = .93). Afterwards, they were debriefed that the tangram task would not take place any more (but was only intended to manipulate power).

#### Experiments 2–5

The general procedure followed Experiment 1, except for (a) using a different power manipulation and (b) implementing manipulations and information sharing measures within the same (business) context.

To manipulate *power*, we implemented power roles in a business setting following colleagues [35]. Participants imagined being manager (*high* power) or assistant (*low* power) in an investment company. The manager’s task was to instruct the assistant and distribute tasks to him/her; the manager position implied evaluating the assistant and deciding about his/her compensation. The assistants’ task was to follow the manager’s instructions and perform tasks provided by the manager; assistants read that the manager would evaluate them and decide about their compensation. Similar to Experiment 1, social power was, thus, implemented as asymmetric outcome control [10]. To make their role more realistic, participants engaged in a few role-matching tasks: They saw a floor plan of their new office and a photograph of their office decoration (high power: a large, opulent single office; low power: a small, simple shared office) and rated the adequateness of their new office for their work (see [35]).

Then, participants were asked to perform a set of typical working tasks. This included preparing and participating in a ‘business meeting’—which in fact comprised the information pooling paradigm. Following general instructions from Experiment 1, as part of the goal manipulation, participants read that their goal for this meeting either was “to perform well” (*task goal*) or “to perform well together as a group” (*group goal*) or “to perform well as a person” (*individual goal*; except in Experiment 2 without individual goal condition).

Participants then completed the same *information sharing paradigm* as in Experiment 1, here embedded in the business simulation context. Participants read that their investment company had recently bought the old monastery, whose monk had buried a treasure in his prospective grave during the time of Reformation. To find the treasure for their company, they needed to combine pieces of information that the monk had distributed to four letters. Again, they learned that one letter was known to all (i.e., *public*). Similar to Experiment 1, the participant possessed a second letter, while two other people owned the remaining third and fourth letter, respectively; these two owners comprised two assistants (*high* power conditions) or one assistant and one manager (*low* power conditions) from other departments of their company. The rest of the paradigm was identical to Experiment 1; again, each letter contained private information known only to its respective owner, and participants needed to share information with the others to solve the task. In preparing for the meeting, participants indicated which of their own pieces of information they wanted to ‘share, withhold, or distort’. The number of critical (i.e., *private-important*) pieces of information, again, served as outcome.

As manipulation checks, participants reported to which extent they had pursued a *group goal* (Experiment 2: “I pursued the goal that someone of the team will find the correct solution”; “I pursued the goal that we jointly find the correct solution”; *r* = .70; Experiments 3–5: “I had the goal to jointly solve the riddle together with the others”). They also indicated how *powerful* they had felt in their role ([36]; 7 items, e.g., 1 = ‘passive’ to 9 = ‘active; ‘submissive’ to ‘dominant’; Experiments 2–5, respectively: αs = .79, .91, .89, and .85); some experiments included further exploratory variables.

## Results

To test our three hypotheses, we conducted three contrast analyses for each *individual* experiment. Each contrast captured the specific prediction as described by the (1) corruptive, (2) compensatory, and (3) selfish effect (Fig 1 above). Means, contrast effects, and effect sizes for each single study are reported in Table 1.

**Table 1:** Means, standard deviations, and effect sizes for pieces of important-private information being shared as a function of power and goal.

Experiment		task goal		group goal		individual goal		hypotheses tests (mean effect sizes *r* and contrast analyses effects)			auxiliary analyses
		low power	high power	low power	high power	low power	high power	*corruptive effect*	*compensatory effect*	*selfish effect*	*group vs*. *task goal for low power*
**1** *N* = 127	*M*	1.71	0.80	1.14	1.50	0.76	1.23	***r* = .24** *F*(1,121) = 7.336, *p* = .008, η^2^ = .057	***r* = .19** *F*(1,121) = 4.398, *p* = .038, η^2^ = .035	***r* = .25** *F*(1,121) = 8.160, *p* = .005, η^2^ = .063	***r* = –.15** *F*(1,121) = 2.937, *p* = .089, η^2^ = .024
	*SD*	1.27	0.77	1.20	1.19	0.89	1.07				
	*n*	21	20	21	22	21	22				
**2** *N* = 123	*M*	1.62	1.15	2.16	1.93	–	–	***r* = .14** *F*(1,119) = 2.419 *p* = .123, η^2^ = .020	***r* = .23** *F*(1,119) = 6.839 *p* = .010, η^2^ = .054	–	***r* = .16** *F*(1,119) = 3.118, *p* = .080, η^2^ = .026
	*SD*	1.29	1.23	1.04	1.17	–	–				
	*n*	29	33	31	30	–	–				
**3** *N* = 185	*M*	1.71	1.37	2.06	2.06	1.18	1.00	***r* = .09** *F*(1,179) = 1.565 *p* = .213, η^2^ = .009	***r* = .19** *F*(1,179) = 6.476 *p* = .012, η^2^ = .035	***r* = .14** *F*(1,179) = 3.620 *p* = .059, η^2^ = .020	***r* = .10** *F*(1,179) = 1.724, *p* = .191, η^2^ = .010
	*SD*	1.13	1.10	0.98	1.18	1.09	0.93				
	*n*	31	30	34	31	28	31				
**4** *N* = 323	*M*	1.43	1.61	1.98	2.00	1.37	1.62	***r* = –.05** *F*(1,317) = 0.709 *p* = .400, η^2^ = .002	***r* = .10** *F*(1,317) = 3.126 *p* = .078, η^2^ = .010	***r* = .01** *F*(1,317) = 0.064 *p* = .801, η^2^ = .000	***r* = .14** *F*(1,317) = 6.380, *p* = .012, η^2^ = .020
	*SD*	1.22	1.16	1.14	1.10	1.14	1.10				
	*n*	54	54	54	54	54	53				
**5** *N* = 547	*M*	1.23	1.35	1.91	1.84	1.03	1.20	***r* = –.03** *F*(1,536) = 0.508 *p* = .476, η^2^ = .001	***r* = .12** *F*(1,536) = 8.434 *p* = .004, η^2^ = .015	***r* = .05** *F*(1,536) = 1.321 *p* = .251, η^2^ = .002	***r* = .17** *F*(1,536) = 15.991, *p* < .001, η^2^ = .029
	*SD*	1.08	1.13	1.14	1.19	1.09	1.23				
	*n*	91	91	90	94	88	88				
meta-analysis								***r* = .03; *p* = .262** 95% CI [-.0634; .1230] (Experiments 1–5)	***r* = .15; *p* < .001** 95% CI [.0544; .2358] (Experiments 1–5)	***r* = .08; *p* = .079** 95% CI [-.0344; .1989] (Experiments 1,3,4,5)	***r* = .12; *p* = .005** 95% CI [.0293; .2126] (Experiments 1–5)

*Note*. *n* = cell size for each experimental condition; *r* = mean effect size for each effect and experiment; conditions being compared in contrast analyses for the *corruptive effect*: high vs. low power for a task goal; *compensatory effect*: group vs. task goal for high power; *selfish effect*: individual vs. task goal for low power; *group vs*. *task goal for low power*: group vs. task goal for low power (analogously to the ‘compensatory effect’ for high power)

Given that our experiments used highly similar procedures, we also tested effects *across* studies using a meta-analytic approach. Here, we first calculated Pearson’s *r* effect sizes for each experiment (see [37,38]; effect size levels for correlation coefficient: small effect: *r* = .1; medium effect: *r* = .3; large effect: *r* = .5). Second, we computed a meta-analysis for every single effect; we did this based on Fishers *z*-transformed effect sizes (see [39]) and computed a weighted *mean r* as effect size indicator for each proposed effect (i.e., weighed by cell size)—to control for the different cell sizes of studies in line with the Schmidt-Hunter method (e.g., [40]). These weighted mean *r*s are also reported in Table 1. This meta-analytic procedure provides the most parsimonious, unequivocal test of the collected evidence for our three predictions and allowed us to examine the robustness of our proposed effects across all data.

### Manipulation checks

#### Power

We compared *subjective power* for high versus low power using contrasts as predictors in a General Linear Model. Results for each individual experiment yielded significant effects, .16 < *r* < .89, .001 < *p* < .028 (see Table 2 for full results).

**Table 2:** Effect sizes for manipulation checks of power- and goal-manipulations.

*Experiment*	*power* *manipulation check*	*goal* *manipulation check*	*additional* *power check*
**1** (*N* = 127)	***r* = .88** *F*(1,121) = 422.987 *p* < .001, η^2^ = .778	***r* = .34** *F*(1,121) = 15.491 *p* < .001, η^2^ = .113	***r* = .05** *F*(1,121) = 0.341 *p* = .560, η^2^ = .003
**2** (*N* = 123)	***r* = .43** *F*(1,118) = 26.424 *p* < .001, η^2^ = .183	***r* = .20** *F*(1,119) = 4.894 *p* = .029, η^2^ = .040	***r* = .05** *F*(1,118) = 0.255 *p* = .615, η^2^ = .002
**3** (*N* = 185)	***r* = .17** *F*(1,174) = 5.003 *p* = .027, η^2^ = .028	***r* = .30** *F*(1,176) = 17.044 *p* < .001, η^2^ = .088	***r* = .01** *F*(1,174) = 0.031 *p* = .861, η^2^ = .000
**4** (*N* = 323)	***r* = .26** *F*(1,317) = 23.861 *p* < .001, η^2^ = .070	***r* = .28** *F*(1,317) = 27.022 *p* < .001, η^2^ = .079	***r* = .00** *F*(1,317) = 0.000 *p* = 1.000, η^2^ = .000
**5** (*N* = 547)	***r* = .39** *F*(1,536) = 97.364 *p* < .001, η^2^ = .154	***r* = .18** *F*(1,534) = 16.948 *p* < .001, η^2^ = .031	***r* = .04** *F*(1,536) = 0.732 *p* = .393, η^2^ = .001
meta-analysis	***r* = .41** *p* < .001 95% CI [.3675; .4579]	***r* = .24** *p* < .001 95% CI [.1881; .2909]	***r* = .03** *p* = .158 95% CI [-.0268; .0822]

*Note*. *r* = mean effect size for each effect and experiment, based on contrast analyses; *power check*: comparing subjective power for the high vs. low power conditions; *group goal check*: comparing subjective group goal pursuit for the group goal vs. task goal (and individual goal) conditions; *additional power check*: comparing subjective power for the high power/task goal vs. high power/group goal condition, this served to make sure that the group (vs. task) goal manipulation did not lower high-power people’s subjective level of power

The follow-up meta-analysis on the effect sizes of these contrasts across experiments also showed a significant effect, *r* = .41, *p* < .001, 95% CI [.3675; .4579]. Accordingly, as intended, high-power conditions induced a greater sense of power than low-power conditions. We, thus, consider the power manipulations successful.

#### Goal

With similar procedures, we compared to which extent participants reported the *pursuit of a group goal* in the group goal compared to the other conditions (i.e., task and individual goal condition; in Experiment 2 only comparing group goal condition to task goal condition). Results for every individual experiment yielded significant effects, .17 < *r* < .35, .001 < *p* < .030 (see Table 2 for full results).

Also, the follow-up meta-analysis across studies yielded an effect, *r* = .24, *p* < .001, 95% CI [.1881; .2909]. As such, participants in the group-goal condition reported that they had pursued a group goal (solving the task together) more so than participants in the other two condition(s), supporting the success of our goal manipulation across experiments.

### Information sharing effects

#### The ‘corruptive effect’

We expected that given a task goal, power-holders share less important-private information than the powerless (Hypothesis 1). Regarding the first three studies we conducted, results supported this prediction for Experiment 1, *r* = .24, *p* = .008, but neither for Experiment 2, *r* = .14, *p* = .123, nor for Experiment 3, *r* = .09, *p* = .213 (see Table 1). Accordingly, results for these first three studies yielded mixed evidence. For our two replication studies, neither Experiment 4, *r* = –.05, *p* = .400, nor Experiment 5, *r* = –.03, *p* = .476, provided support for this prediction.

Moreover, analyzing data for the five experiments meta-analytically yielded no evidence for a corruptive effect, *r* = .03, *p* = .262, 95% CI [-.0634; .1230]. Hence, overall, data did not support that there may be a corruptive effect of power on information sharing.

#### The ‘compensatory effect’

Furthermore, we predicted that a group goal (rather than task goal) compensates for power-holders’ low information sharing (Hypothesis 2). Separate analyses for each experiment yielded support for this effect across the first three studies, namely, Experiment 1, *r* = .19, *p* = .038, Experiment 2, *r* = .23, *p* = .010, and Experiment 3, *r* = .19, *p* = .012. Also, the two replication studies supported this prediction, Experiment 4 marginally, *r* = .10, *p* = .078, and Experiment 5, *r* = .12, *p* = .004 (again, see Table 1).

Furthermore, the meta-analysis of all five experiments yielded a small, but significant effect, *r* = .15, *p* < .001, 95% CI [.0544; .2358]—suggesting that high-power people shared more important-private information when given a group than a task goal (but see also auxiliary analyses below). Importantly, this ‘compensatory effect’ was not due to a group goal reducing people’s subjective sense of power: Across studies, high-power people did experience a *similar* amount of power in the task- and the group-goal condition, *r* = .038, *p* = .158, 95% CI -.0268; .0822], see additional power checks in Table 2. Hence, a changed *sense of power* in the group goal condition cannot explain such an effect.

#### The ‘selfish effect’

Finally, we assumed that low-power people share less critical information when given an individual goal (compared to a task goal; Hypothesis 3). Separate analyses for the first three experiments yielded support only in Experiment 1, *r* = .25, *p* = .005, and marginally in Experiment 3, *r* = .14, *p* = .059 (Experiment 2 did not examine this effect). However, our two replication studies did not support this prediction, Experiment 4, *r* = .01, *p* = .801, and Experiment 5, *r* = .05, *p* = .251 (see Table 1).

Mirroring these mixed results, a meta-analysis across these four studies showed only a very small, marginal effect, *r* = .08, *p* = .079, 95% CI [-.0344; .1989]. Accordingly, there was some weak evidence suggesting a selfish effect of an individual (vs. task) goal among the powerless, but this remains largely tentative.

### Summary and auxiliary analyses for low power

In sum, results yielded weak to no evidence for our predictions: Power-holders did not seem to share less information than the powerless under a task goal (i.e., no evidence for a corruptive effect). A group goal seemed to promote information sharing among the powerful: Power-holders shared more important information with others when pursuing a group rather than a task goal (a small-to-medium compensatory effect). With regard to this ‘compensatory effect’ of a task versus group goal among the powerful, however, data suggested that this effect may not be specific to and/or greater for the powerful, but may also apply to the *powerless* (see Means in Table 1).

Accordingly, we performed auxiliary analyses—testing if a task versus group goal also enhanced information sharing among those *low* in power. Separate analyses for each experiment yielded (descriptive) support for such an effect among the powerless (Experiment 2, *r* = .16, *p* = .080, Experiment 3, *r* = .10, *p* = .191, Experiment 4, *r* = .14, *p* = .012, Experiment 5, *r* = .17, *p* < .001) with the exception of Experiment 1 (*r* = –.15, *p* = .0890; see Table 1 last column). Furthermore, a meta-analysis of all five experiments yielded a small, but significant effect, *r* = .12, *p* = .005, 95% CI [.0293; .2126]—indicating that also low-power people (just as high-power people) shared more important-private information when given a group goal, rather than a task goal. Notably, this effect size for low-power people (*r* = .12) is highly similar to the mean effect size for the compensatory effect for high-power people (*r* = .15), and Confidence Intervals for these two effects largely overlap.

Taken together, this suggests that in total, there is a benefit of a group (versus task) goal promoting information sharing—but *both* among the powerful and the powerless. This means that we did not find support for a specific ‘compensatory effect’ among the powerful (also given that there is no corruptive effect to compensate in the first place), but rather a beneficial effect of a group versus task goal on information sharing (irrespective of power).

Finally, our hypotheses tests suggested that the powerless might to some extent withhold critical information when pursuing an individual goal (rather than a task goal; ‘selfish effect’); this marginal effect, however, needs to be interpreted cautiously. Given that we did not find evidence for the corruptive effect and very limited evidence for the selfish effect across studies, we only report results for the ‘mediation effect’ (i.e., that selfish motivation may explain effects of Power and Goal on information sharing) in S1 Supporting Information.

## Discussion

Team members making decisions together often have access to unique, important pieces of information that only they possess. When shared with their fellow members, these pieces of information can boost the group’s decision-quality and performance [41]. Yet, team members, per default, often seem to follow an individual goal to ‘solve the task alone’—which brings them to withhold information [2]. Investigating the role of high (and low) power within groups, the present research examined (a) if especially power-holders may selfishly withhold such information and (b) if a group goal can help overcoming this.

We started with the idea that power-holders, often behaving more selfishly than the powerless, would (1) refrain from sharing information—given “standard” conditions, namely, a task goal. Following up on this, (2) a goal compensating for this selfishness would motivate the powerful to share their information—namely, in case of a group (vs. task) goal. Finally, (3) motivating the powerless (who usually are selfless) to behave selfishly would lower their information sharing—in case of an individual (vs. task) goal.

We conducted and reported five experiments to test this. Meta-analytical evidence across all these experiments, including two highly-powered replications and different power manipulations, yielded weak to no support for our predictions. Specifically, results across studies yielded no evidence for (1) the corruptive effect and very weak evidence for (3) the selfish effect; we found evidence for the benefits of a group versus task goal (as included in the predicted (2) compensatory effect); but here, auxiliary analyses suggested that, first, this effect is not only true for high, but also for low power, and second, that this effect may need to be labelled differently (since there was no evidence for a corruptive effect to be ‘compensated’). Interpreting this overall evidence, our results do not support a *power effect* (i.e., that social power might alter information sharing), but only support a *goal effect* (i.e., that goals do alter information sharing). We discuss these two aspects in the following.

### Power effects: No corruptive effect of power?

Prior research indicates that power does often tempt people to focus on personal interests, opinions, or benefits (e.g., [14, 15]) and to distrust others’ contributions (e.g., others’ advice; [17, 18]; or others’ offers for a favor; [42])—which, in the long-run, is likely to hinder interpersonal relationships and cooperation. As such, power often seems to promote selfishness—which we assumed may also imply sharing less important-private information with others (under specific goal conditions). Yet, our lack of evidence for the corruptive effect of power suggests that either there is no effect of power on information sharing, or that such an effect may depend on (other) moderators (e.g., only occur under specific conditions not implemented here). It could be possible that we did not find evidence for an effect of power because, even under a standard *task goal*, power may not always lead to selfish behavior. We outline potential explanations below, which at this point are largely speculative.

On potential moderator could be the *power context* implemented. Experiment 1 seemed the only study yielding some support for a corruptive effect of power; considering its low sample size, this finding needs to be interpreted with caution. Nonetheless, when cautiously examining this study, power was manipulated in a context *un*related to the information sharing context—as is often the case in power research. This paradigm allows for testing “mindset” effects of power priming that are based on cognitive tuning towards specific behavior [43]. Accordingly, such a paradigm controls for context or demand effects and reveals predominantly social cognitive effects. Experiment 1 using such a paradigm seemed to support a potential corruptive effect.

In contrast, the other four experiments tested power and information sharing in the same hierarchical context; here, we found very weak to no evidence for selfish behavior among the powerful. This could point towards certain demands and role expectations people may have within a power context: Followers may expect their power-holder to act as a role model and to share important knowledge. When followers are part of the team (i.e., when given power in the same context), power-holders might act in line with these expectations. When followers come from a different context and power-holders are liberated from such role expectations (i.e., when given power in another context), power-holders might be more prone to follow a selfish agenda and keep information to themselves. This idea is certainly tentative and post-hoc; however, since we only conducted one priming study, more research is needed to differentiate between such potential carry-over and context-sensitive effects of power.

A second potential moderator could be the *level of selfishness*, as reflected by specific outcomes of power being investigated. Our research tested effects on information sharing. Withholding important, unique pieces of information likely constitutes a selfish tendency, because doing so keeps others (e.g., the team) from finding a correct solution; yet, other behavioral outcomes might reflect even stronger selfish tendencies, such as keeping crucial financial resources to oneself or using up more of those for the self. It could be that power produces stronger effects (only) on such outcomes, rather than on information sharing.

Finally, the *meaning* that people associate with power could moderate potential effects of power. Indeed, an increasing body of evidence has shown that power can promote prosocial (rather than selfish) tendencies. At times, especially the powerful try to ensure good *team* outcomes [8], treat others *fairly* [44], and carefully *individuate* others [45], *value* others’ advice [46], or even *forgive* others a personal insult [47]. Accordingly, power can promote selfless behavior—which may be another reason why we did not find support for a corruptive effect of power. One approach to explain when power heightens or lowers selfishness under “standard” (task goal) conditions is that people can construe (i.e., appraise) power differently [28,37]. People sometimes construe high power as an opportunity to “make things happen”—which invites more selfishness towards individual goals and personal benefits; yet, at other times, people construe power as responsibility to “take care of things”—which may rather invite consideration of others (e.g., [28,46]).

Our studies did not induce a specific construal of power; yet, it is still possible that power-holders in our standard “task goal” condition construed power differently. It may be that some construed their power as an opportunity (thus, keeping more information to themselves), whereas others construed power as responsibility (thus, sharing more information); if so, these opposing effects could have resulted in lack of evidence for a corruptive effect. This assumption, however, needs to be tested in future research. As power research often targets such “standard” (task goal) conditions, investigating this would foster an understanding of when power may (not) induce selfishness in “standard” situations.

In line with this idea, some initial results from Larson and colleagues [7,48] suggested that especially the leader plays an active role in repeating information in discussions in medical teams. In their studies, the leader was *accountable* for the team outcome (the authors did not focus on the role of power *without* accountability, i.e., our standard task goal context). We reason it might be that due to their accountability, leaders here construed their power-position more as responsibility—which may explain why they engaged so constructively in the group discussion (in that study not by sharing, but by asking questions in the team).

To conclude, our data did not support a potential corruptive effect of power on information sharing. This suggests either that there may not be such an effect of power or that a potential corruptive effect of power may only occur under specific conditions not investigated here (e.g., in case of carry-over effects or a specific construal of power). These possibilities outline avenues for future research about power, goals, information sharing, and other potential (selfish) outcomes.

### Goal effects: Benefits of a group goal?

Our experiments, taken together, seem to yield the clearest evidence in favor of an effect of the *goal* people received: Indeed, data suggests that a *group goal* (compared to a task or individual goal) fostered information sharing with others. This finding is in line with prior evidence that group goals provide less room for selfish tendencies—such as enhance perceived cooperation within a group [20], enhance information seeking with fellow group members [21], and motivate members to consider each other’s information [22].

Going beyond this, the sum of evidence in our studies indicates that this beneficial effect of a group (compared to other) goal(s) is likely relatively *independent* of people’s level of power in the group. Accordingly, it may be that the best way to ensure successful cooperation across (or within) hierarchies is to promote group goals among team members (irrespective of their hierarchical position).

This has important practical implications. Considering the high relevance of information sharing within so-called knowledge work [49], many organizations search for ways how to manage their members’ knowledge. Indeed, both the leader and team members may possess unique information that, if being shared, can improve decision quality and team performance. From an organizational perspective, it often matters more *that* the best solution is identified (i.e., that team members do share their information), rather than *who* identifies it. Our findings open possibilities for interventions aiming at a more open communication in hierarchies. Promoting (shared) group goals in an organization—for example, during regular meetings or on internal communication platforms—should enhance leaders’ and followers’ willingness to share relevant information. In this sense, organizations already implementing these aspects should have less information sharing problems.

To conclude, many important decisions are left for teams to make—because combining each person’s unique information can help to optimize a team’s decision. Yet, team members sometimes selfishly keep information from others, rather than trying to find the best solution together. To make sure that “those who know do share”, it seems most important to highlight the necessity to *work together* in finding the solution—in short, to promote group goals (more so than consider each member’s power). This outlines a fruitful starting point to foster information sharing in hierarchies.

## Supporting information

S1 Supporting Information Mediation analyses testing the mediation effect. (DOCX)

S2 Supporting Information Items assessing selfish motivation in Experiment 2. (DOCX)

S3 Supporting Information Preregistration of Experiment 4. (PDF)

S4 Supporting Information Preregistration of Experiment 5. (PDF)
