# Supplementary material for: When those who know do share: Group goals facilitate information sharing, but social power does not undermine it
Source: PLoS One. 2019 Mar 11;14(3):e0213795. doi: 10.1371/journal.pone.0213795 (PMC6411119; doi:10.1371/journal.pone.0213795)
Supplement: S1 Supporting Information — (DOCX) [file pone.0213795.s001.docx]

**S1. Supporting information.** Items assessing selfish motivation in Experiment 2

We originally expected that (4) the effects of Power and Goal on information sharing would be mediated by higher selfish motivation (‘mediation effect’); analyses for this mediation effect—which was tested in Experiments 2 and 3—are reported below. Please note, however, that we did not find evidence for the ‘corruptive effect’ and very limited evidence for the ‘selfish effect’ across studies; moreover, data suggested that the ‘compensatory effect’ was not specific to high power, but also applied to low power. Accordingly, mediation analyses reported below need to be interpreted cautiously and with an awareness of this lack of evidence for the predicted effects on information sharing across our five experiments.

**Measures**

***Selfish motivation.*** In Experiments 2 and 3, we additionally assessed selfish motivation. *Experiment 2* included 20 items related to participants’ motivation to share information (see S2 Supporting Information)—established to address self-/other-oriented motivation in information exchange [36] and power research [37]. All items used scales from 1 (*does not apply at all*) to 9 (*completely applies*).

Because these items were from two separate domains of research, we conducted a factor analysis.^1^ It revealed a first factor, depicting *self-*oriented motivation (9 items; e.g., “I had the wish to be the first one solving the riddle”, “I sought to be the only one knowing the solution”; Cronbach’s α = .90), and a second factor, representing *group-*oriented motivation (7 items; e.g., “I cared about making a contribution to identifying the solution”, “I made an effort that the meeting proceeded successfully”; Cronbach’s α = .92). The remaining four items were difficult to interpret as theoretical factor and excluded from analyses. The two factors were highly correlated, *r*(123) = –.63, *p* < .001, and thus combined into a composite score for *selfish motivation* (group-oriented items reversely coded).

In *Experiment 3*, we used short versions of the self-oriented (6 items; α = .94) and group-oriented motivation from Experiment 2 (5 items; α = .86; 1–strongly disagree to 7–strongly agree). Again, both scales were highly correlated, *r*(*182*) = -.39, *p* < .001, and combined to represent *selfish motivation*. In addition, we explored if participants perceived all people involved in the meeting as being either one group or separate individuals (two items on the extent of either of those options; 1 = not at all, to 7 = very much; *r* = –.86; see [38]). We pooled these two items together as one exploratory *self-centered perception* score. ^2^

**Analyses**

***The mediation effect via selfish motivation (Experiments 2 and 3).*** We tested whether (measured) selfish motivation explains potential differences in information sharing across Power and Goal conditions (Hypothesis 4). In a first step, we translated our three predicted Power and Goal effects into a priori contrasts.

In Experiment 2, the condition high power / task goal should reduce information sharing and enhance selfish motivation, compared to low power / task goal, low power / group goal, and high power / group goal. The corresponding contrast (-3 +1 +1 +1) was significant for information sharing (i.e., the total effect), *F* (1, 119) = 9.76, *p* = .002, *η^2^_part_*_._ = .076, as well as for the mediator selfish motivation, *F* (1, 119) = 7.33, *p* = .006, *η^2^_part_*_._ = .061—high power with a task goal lowered information sharing and enhanced selfish motivation compared to the other conditions in this specific study.

In Experiment 3, our hypotheses, together, predicted that not only high power / task goal, but also both high and low power / individual goal conditions would result in low information sharing (all coded as -1): For low power, an individual goal should induce selfishness; for high power, an individual goal should have the same (selfish) effect as a nonspecific task goal. The contrast capturing this overall prediction (-1 +1 +1 +1 -1 -1) was significant for information sharing, *F* (1, 176) = 22.00, *p* < .001, *η^2^_part_*_._ = .111, and for the mediator selfish motivation, *F* (1, 176) = 24.66, *p* < .001, *η^2^_part_*_._ = .123.

Moreover, in Experiment 3, we measured to which extent participants perceived the people involved in the meeting as separate individuals, rather than as a group. The a priori contrast (+1 -1 -1 -1 +1 +1) analysis for this *self-centered perception* as outcome was significant in this study, *F* (1, 176) = 20.28, *p* < .001, *η^2^_part_*_._ = .103. Participants in the high power / task goal condition and in the high power / individual goal and low power / individual goal conditions *perceived* the meeting in a more self-centered manner than all other conditions.

**Mediation analyses*.*** In a second step, we applied two mediation analyses with the bootstrapping macro (Hayes, 2009), see Fig S2. For Experiment 2, we entered the a priori contrast (-3 +1 +1 +1) as independent variable, selfish motivation as mediator, the number of shared important-private pieces of information as dependent variable, and two orthogonal contrasts capturing the residual variance as covariates. The indirect effect was significant in this study, *B* = .161, *SE* = .049, CI 95% = [.0594; .2509]. The contrast predicted selfish motivation, which in turn predicted less information sharing, *B* = -.479, *SE* = .037, *p* < .001.

For Experiment 3, we ran a similar mediation analysis with the respective contrast (-1 +1 +1 +1 -1 -1) as independent variable, selfish perception as additional (parallel) mediator, and four orthogonal contrasts capturing all residual variance as covariates. The indirect effect was significant for the mediator *selfish motivation*, *B* = .260, *SE* = .055, CI 95% = [.1613; .3784], but not for the mediator *self-centered perception*, *B* = −.004, *SE* = .029, CI 95% = [−.0629; +.0530], see Fig S2. This suggests that, in this study, being *selfishly* *motivated* (in the high power / task goal condition) reduces sharing of critical information, whereas perceiving the situation from a more self-centered standpoint is not driving this effect.

*
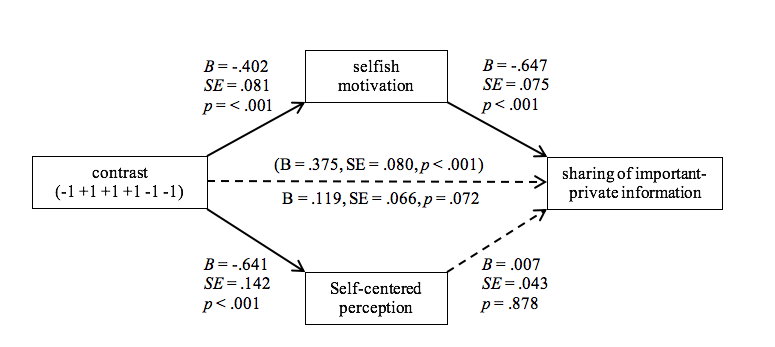
*

**Fig S2. Mediation model.** Two-mediator model for the effects of the a priori contrast (for high power / task goal, low power / task goal, high power / group goal, low power / group goal, high power / individual goal, low power / individual goal, respectively) on sharing of important-private information in Experiment 3. The total effect is displayed in parentheses. Dashed lines represent non-significant paths in the full model, solid lines represent significant paths.

**Footnotes**

1: An exploratory factor analysis (principal components) was conducted to identify the items that best suited as a composite score of selfish motivation. The first three factors explained 42%, 14%, and 7% of the variance, respectively – and after these factors the eigenvalues were ‘leveling off’ in the scree plot. Accordingly, a three-factor solution was examined using Varimax rotation of the factor loadings.

2: Three participants did not finish the motivational measures (remaining N = 182). For exploratory reasons, we assessed public self-awareness (6 items) and accountability (3 items) at the end of Experiment 3, as well as perceived opportunities (10 items) and responsibility (13 items), followed by a hypothetical lottery ticket distribution, at the end of Experiment 1. These exploratory measures (assessed after the main dependent measures) were exploratory and are not considered further here
