# Supplementary material for: When those who know do share: Group goals facilitate information sharing, but social power does not undermine it
Source: PLoS One. 2019 Mar 11;14(3):e0213795. doi: 10.1371/journal.pone.0213795 (PMC6411119; doi:10.1371/journal.pone.0213795)

**S2. Supporting information.** Items assessing selfish motivation in Experiment 2

[*Original version in German; For English translation see below*]

Bevor Sie das Rätsel lösen, bitten wir Sie noch ein paar Fragen zu beantworten, die Ihre gerade getroffenen Entscheidungen zur Vorbereitung des Meetings betreffen.

Geben Sie bitte spontan an, wie die folgenden Aussagen auf Sie zutreffen:

| **In Bezug auf meine Entscheidungen...** | trifft gar  nicht zu |  | trifft voll- ständig zu |
| --- | --- | --- | --- |
| habe ich mich darum gekümmert, meinen Teil zur Lösung beizutragen. | ➀ ➁ ➂ ➃ ➄ ➅ ➆ ➇ ➈ | | |
| fühlte ich mich verantwortlich, dass die Lösung gefunden wird. | ➀ ➁ ➂ ➃ ➄ ➅ ➆ ➇ ➈ | | |
| habe ich mich bemüht, dass das Meeting erfolgreich verläuft. | ➀ ➁ ➂ ➃ ➄ ➅ ➆ ➇ ➈ | | |
| war ich verantwortlich, zum Meeting beizutragen. | ➀ ➁ ➂ ➃ ➄ ➅ ➆ ➇ ➈ | | |
| war ich schuld, wenn die Dinge im Meeting schlecht verlaufen. | ➀ ➁ ➂ ➃ ➄ ➅ ➆ ➇ ➈ | | |
| habe ich versucht, ehrlich zu sein. | ➀ ➁ ➂ ➃ ➄ ➅ ➆ ➇ ➈ | | |
| habe ich versucht zu vermeiden, dass ich ausgenutzt werde. | ➀ ➁ ➂ ➃ ➄ ➅ ➆ ➇ ➈ | | |
| habe ich versucht, meine eigenen Chancen auf die Lösung zu maximieren. | ➀ ➁ ➂ ➃ ➄ ➅ ➆ ➇ ➈ | | |
| wollte ich das Rätsel lösen, um einen persönlichen Erfolg zu haben. | ➀ ➁ ➂ ➃ ➄ ➅ ➆ ➇ ➈ | | |
| hätte die Lösung des Rätsels gerne als einzige/r gewusst. | ➀ ➁ ➂ ➃ ➄ ➅ ➆ ➇ ➈ | | |
| hatte ich den Wunsch, das Rätsel als erster zu lösen. | ➀ ➁ ➂ ➃ ➄ ➅ ➆ ➇ ➈ | | |
| wollte ich den anderen eine „Lektion“ erteilen. | ➀ ➁ ➂ ➃ ➄ ➅ ➆ ➇ ➈ | | |
| habe ich das Ziel verfolgt, dass nur ich selbst die richtige Lösung finde. | ➀ ➁ ➂ ➃ ➄ ➅ ➆ ➇ ➈ | | |
| habe ich unabhängig von den anderen das Ziel verfolgt, die richtige Lösung zu finden. | ➀ ➁ ➂ ➃ ➄ ➅ ➆ ➇ ➈ | | |
| konnte ich die Möglichkeiten sehen, die sich mir bieten. [dropped] | ➀ ➁ ➂ ➃ ➄ ➅ ➆ ➇ ➈ | | |
| hatte ich mehr Möglichkeiten, um meine Ziele zu erreichen. | ➀ ➁ ➂ ➃ ➄ ➅ ➆ ➇ ➈ | | |
| habe ich Gelegenheiten genutzt, um erfolgreich zu sein. [dropped] | ➀ ➁ ➂ ➃ ➄ ➅ ➆ ➇ ➈ | | |
| hatte ich Kontrolle. [dropped] | ➀ ➁ ➂ ➃ ➄ ➅ ➆ ➇ ➈ | | |
| konnte ich meinen eigenen Anliegen folgen. [dropped] | ➀ ➁ ➂ ➃ ➄ ➅ ➆ ➇ ➈ | | |

[*English translation*]

*Bevor you proceed solving the mystery, please answer some questions that are related to your decisions you just made for preparing the work meeting.*

*Please indicate spontaneously how the following statements apply to you:*

| **With regard to my decisions...** | does not apply  at all |  | completely  applies |
| --- | --- | --- | --- |
| I cared about making my contribution to identifying the solution. | ➀ ➁ ➂ ➃ ➄ ➅ ➆ ➇ ➈ | | |
| I felt responsible that the solution will be found. | ➀ ➁ ➂ ➃ ➄ ➅ ➆ ➇ ➈ | | |
| I made great efforts that the meeting will be successful. | ➀ ➁ ➂ ➃ ➄ ➅ ➆ ➇ ➈ | | |
| I was responsible for contributing to the meeting. | ➀ ➁ ➂ ➃ ➄ ➅ ➆ ➇ ➈ | | |
| it was my fault if the meeting is not successful. | ➀ ➁ ➂ ➃ ➄ ➅ ➆ ➇ ➈ | | |
| I tried to be honest. | ➀ ➁ ➂ ➃ ➄ ➅ ➆ ➇ ➈ | | |
| I tried to avoid being exploited. | ➀ ➁ ➂ ➃ ➄ ➅ ➆ ➇ ➈ | | |
| I aimed to enhance my own chances for finding the solution. | ➀ ➁ ➂ ➃ ➄ ➅ ➆ ➇ ➈ | | |
| I wanted to solve the riddle to have a personal success. | ➀ ➁ ➂ ➃ ➄ ➅ ➆ ➇ ➈ | | |
| I sought to be the only one knowing the solution. | ➀ ➁ ➂ ➃ ➄ ➅ ➆ ➇ ➈ | | |
| I had the wish to be the first one solving the riddle. | ➀ ➁ ➂ ➃ ➄ ➅ ➆ ➇ ➈ | | |
| I wanted to teach the others a lesson. | ➀ ➁ ➂ ➃ ➄ ➅ ➆ ➇ ➈ | | |
| I was striving for the goal that only I will find the correct solution. | ➀ ➁ ➂ ➃ ➄ ➅ ➆ ➇ ➈ | | |
| I was independently from the others striving for the goal to find the correct solution. | ➀ ➁ ➂ ➃ ➄ ➅ ➆ ➇ ➈ | | |
| I could see the opportunities that presented themselves to me. [dropped] | ➀ ➁ ➂ ➃ ➄ ➅ ➆ ➇ ➈ | | |
| I had more opportunities to reach my goals. | ➀ ➁ ➂ ➃ ➄ ➅ ➆ ➇ ➈ | | |
| I was taking the opportunity to be successful. [dropped] | ➀ ➁ ➂ ➃ ➄ ➅ ➆ ➇ ➈ | | |
| I had control. [dropped] | ➀ ➁ ➂ ➃ ➄ ➅ ➆ ➇ ➈ | | |
| I could follow my own concerns. [dropped] | ➀ ➁ ➂ ➃ ➄ ➅ ➆ ➇ ➈ | | |

Bitte kreuzen Sie im Folgenden noch die Darstellung an, die am besten Ihre Nähe zu den anderen beiden Briefbesitzern beschreibt:

*Please choose the picture that, in your opinion, best represents how close you are to the two other letter owners:*

[*see circle measure below: with the label “myself“ on the left and ”other letter owners” on the right*]


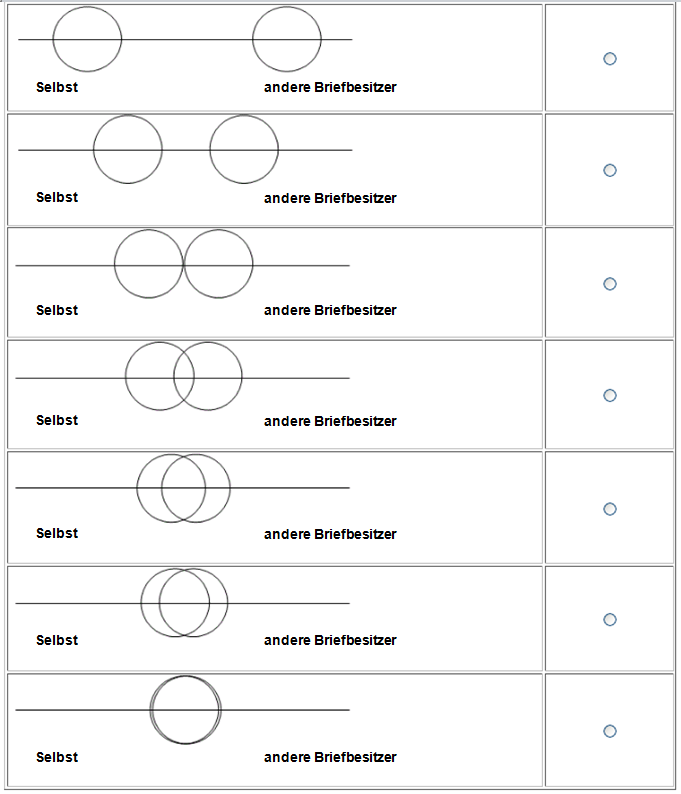

Supplement: S2 Supporting Information — (DOCX) [file pone.0213795.s002.docx]
