# Supplementary material for: When those who know do share: Group goals facilitate information sharing, but social power does not undermine it
Source: PLoS One. 2019 Mar 11;14(3):e0213795. doi: 10.1371/journal.pone.0213795 (PMC6411119; doi:10.1371/journal.pone.0213795)
Supplement: S3 Supporting Information — (PDF) [file pone.0213795.s003.pdf]

S3. Supporting Information. Preregistration of Experiment 4.

As Predicted:"*I4 - power info exchange replication mturk*" (#5058)

Created: 08/03/2017 01:40 AM (PT)

Author(s)
Annika Scholl (Leibniz-Institut für Wissensmedien) - a.scholl@iwm-tuebingen.de

1) What's the main question being asked or hypothesis being tested in this study?
We test the following specific predictions: (1) In case of a task goal, power-holders should share less information than those low in power ('corruptive effect' of power); (2) A grou (versus task) goal, however, should compensate this effect and promote information sharing among the—usually more selfish—power-holders ('compensatory effect'); (3) Similar individual (versus task) goal may increase selfishness among the—usually less selfish—powerless and lower their information sharing ('selfish effect').

2) Describe the key dependent variable(s) specifying how they will be measured.
information sharing (unshared, important pieces of information) in information pooling game

3) How many and which conditions will participants be assigned to?
2 (power: low vs. high) x 3 (goal:task vs. group vs. individual)
between conditions, random assignment via qualtrics randomizer

4) Specify exactly which analyses you will conduct to examine the main question/hypothesis.
We test the specific predictions (1) via contrasts and (2) meta-analytically combined with 3 prior studies.

5) Any secondary analyses?
2 x 3 ANOVA

6) How many observations will be collected or what will determine sample size?
No need to justify decision, but be precise about exactly how the number will be determined.
determined via g-power and effect sizes from the previous studies:
mean r (3 studies) = .18
equals effect size f = .183 (see http://www.psychometrica.de/effect_size.html#transform), alpha .05, power .90, 1 df, 6 groups, fixed affects ANOVA with interactions
ideal N = 316

7) Anything else you would like to pre-register?
(e.g., data exclusions, variables collected for exploratory purposes, unusual analyses planned?)
Requirements for participation:
- ps read all information carefully (reasonable completion time)
- unfamiliar with materials and unsuspicious of hypothesis (prevent demand effects)
- fluent in English (language-sensitive materials)

Any data from ps who wish to withdraw their data after debriefing will be deleted (see ethical guidelines).

8) Have any data been collected for this study already?
No, no data have been collected for this study yet

MAKE PUBLIC
