# Supplementary material for: When those who know do share: Group goals facilitate information sharing, but social power does not undermine it
Source: PLoS One. 2019 Mar 11;14(3):e0213795. doi: 10.1371/journal.pone.0213795 (PMC6411119; doi:10.1371/journal.pone.0213795)
Supplement: S4 Supporting Information — (PDF) [file pone.0213795.s004.pdf]

S4. Supporting Information. Preregistration of Experiment 5.

You are logged in as: **a.scholl@iwm-tuebingen.de** (Log out (logou

HOME (index.php)
BACK (see_list.php)

Make Sugg
(mailto:larry@AsPredicted.org?Sul
have a suggestion for AsPre
Change my email (update_email

As Predicted:"*I5 - power info exchange replication uni-rundmail*" (#6840)

Created: 11/20/2017 12:32 AM (PT)

Author(s)
Annika Scholl (Leibniz-Institut für Wissensmedien) - a.scholl@iwm-tuebingen.de

1) Have any data been collected for this study already?
No, no data have been collected for this study yet.

2) What's the main question being asked or hypothesis being tested in this study?
We test the following specific predictions: (1) In case of a task goal, power-holders should share less information than those low in power ('corruptive effect' of power); (2) A grou (versus task) goal, however, should compensate this effect and promote information sharing among the—usually more selfish—power-holders ('compensatory effect'); (3) Similar individual (versus task) goal may increase selfishness among the—usually less selfish—powerless and lower their information sharing ('selfish effect').

3) Describe the key dependent variable(s) specifying how they will be measured.
information sharing (unshared, important pieces of information) in information pooling game

4) How many and which conditions will participants be assigned to?
2 (power: low vs. high) x 3 (goal:task vs. group vs. individual)
between conditions, random assignment via qualtrics randomizer

5) Specify exactly which analyses you will conduct to examine the main question/hypothesis.
We test our specific predictions & hypotheses (1) via contrasts and (2) meta-analytically combined with 3 prior studies.
Potential 2 x 3 ANOVA as a follow-up

6) Describe exactly how outliers will be defined and handled, and your precise rule(s) for excluding observations.
Requirements for participation:
- ps read all information carefully (reasonable completion time)
- unfamiliar with materials and unsuspicious of hypothesis (prevent demand effects)
- fluent in German (language-sensitive materials)
- aged 18-35 years (materials designed for "typical" undergraduate students)
Any data from ps who wish to withdraw their data after debriefing will be deleted (see ethical guidelines). We will carefully check (& report) if excluding ps not fulfilling the criteria changes results.

7) How many observations will be collected or what will determine sample size?
No need to justify decision, but be precise about <u>exactly</u> how the number will be determined.
determined via g-power and effect sizes from the previous studies: mean r (3 studies) = .18; equals effect size f = .183 (see http://www.psychometrica.de/effect_size.html#transf alpha .05, power .90, 1 df, 6 groups, fixed affects ANOVA with interactions; ideal N = 316; we still consider this may be optimistically calculated, which is why we aim at an ideal N=380 (provided that as many ps can be recruited; after that or after 1.5 weeks, data collection will be stopped)

8) Anything else you would like to pre-register?
(e.g., secondary analyses, variables collected for exploratory purposes, unusual analyses planned?)
Potential 2 x 3 ANOVA as a follow-up

Recall: Approved pre-registrations remain private until a participating author acts to make it public
APPROVE REJECT
Want to make changes? (messages/edit.php)
